# Mr. Hill's Cases of Inoculation

**Published:** 1806-08

**Authors:** Samuel Hill

**Affiliations:** 158, Queen Street, Town of Portsea, Hants


### *To the Editors of the Medical and Physical Journal.*

GENTLEMEN,

SOME cases having lately occurred in this vicinity, which have been industriously circulated as cases of small-pox subsequent to vaccination, I beg you will oblige me by inserting the following statement of facts in the Medical and Physical Journal for the ensuing month, lest they should, if not contradicted by me, obtain general credit as such.

The first is a case of small-pox eight years and three months after the patient had been variolated.—On the 19th of March, 1798, I variolated a child of Mr. Creswells, Half-way Houses, Portsea, then about three months old. The arm rose and there was constitutional affection, altho' no eruptions appeared. I however (as well as the parents) was perfectly satisfied that the child was secure; if I had had any doubt respecting it, I most certainly would not have received the reward until I had by repeated inoculation placed their child in safety. On the 13th of June last, Mr. Cooper, a respectable practitioner, at the Half-way Houses, Portsea, was so friendly as to inform me, that he had been called to Mr. Creswell's child, who after having a smart fever for three days, was relieved by a plentiful crop of eruptions, which he had no doubt would turn out to be small-pox. He was also kind enough to tell me, that Mrs. Cresswell had spread a story abroad, that I had on the date above-mentioned, (March 19, 1798) *imposed* on her by substituting the vaccine for small-pox inoculation, and that she told this story to every person she conversed with. Mr. Cooper told her, that when her child was inoculated, vaccination had not been introduced into this neighbourhood, but she would not be convinced. On the following day (14th ult.) I called on her to have some conversation, and see her child, who certainly had the small-pox pretty full; in my endeavours to convince her that her child had never had the cow-pox, and that I valued my character too much to abuse the confidence my friends had in me, by substituting one inoculation for another, I had as little success as Mr. Cooper; she said, "You have certainly given cow-pox in lieu of small-pox, or my child could not have small-pox now, and *do not try, for I never will be persuaded of the contrary.*" It was useless to say any thing more on the subject, I therefore took my leave.

REMARK

REMARK ON THE PRECEDING.

It may be seen in my Pamphlet, reviewed in your Journal for October, 1804, No. 69, p. 472, that I commenced vaccine inoculation Dec. 5, 1800; I request your readers to compare this latter date with that on which little Cresswell was variolated, and it will be immediately seen, that I could not possibly (if inclined) have committed the fraud Mrs. C. charges me with, as her child was variolated one year and nine months before I began vaccine inoculation.

The next cases stated to be failures of the vaccine inoculation, are the two Mervins of Queen Street, Portsea.

These cases were stated in a letter to Dr. Jenner, when they occurred, and I will take the liberty of extracting from a copy of the same now before me, so much as relates to them. I trust on the present occasion, Dr. Jenner will pardon me for making public, part of any letter I have had the honour of writing to him.

Mr. Mervin was requested by me in June, 1805, to allow me to vaccinate his three children, neither of whom had passed the small-pox; but he had been so decidedly set against vaccination, by the parents of a child, who it was supposed, had taken small-pox subsequently; and as the book* giving an account of that child's case was given to Mervin to read, it had such an effect on the minds of himself and his wife, that they would not consent, altho' it was to have been done gratuitously.

In January last, Mr. Mervin called at my house late in the evening, and requested me to see his eldest child, Edward, on whom the small-pox had appeared four days. The child had sickened seven days before. On seeing him I found him very full of the confluent kind; and indeed, from the symptoms and general appearance, I had but little hope of his recovery.† The two younger children, William and Eliza, having been so long exposed to the variolous contagion, it was reasonable to suppose they would not escape; I therefore again proposed vaccination to the parents, stating, that I did not expect to prevent the small-pox; but that I thought, if it did not prevent it, the

---

* Cases of Small-pox subsequent to Vaccination, published at Portsea, 1804.

† He recovered, is much seamed; it was not till the 21st day, that all the pox had turned.

the complaint might be rendered much milder than Edward's. They consented, and I vaccinated them* immediately with matter received the same day from the Central House, Salisbury Square, having no recent or fluid matter in my possession.

The girl's arm, (one only rose) was very slow in its progress; the vesicle, areola, and subsequent eschar much smaller than I ever saw in a perfect vaccination. On William, I could not succeed in my attempt to give him the Cow-pox; and he was re-vaccinated on the eighth day from his sister, who was also re-vaccinated from her own arm, but without effect. William's second inoculation, proceeded very hastily, and on the fourth day from it, he became restless, and had a slight degree of fever. In two days more, (6th from the latter inoculation) about one hundred eruptions appeared, about eight of which number maturated on his face.

Eliza became dull and heavy on the 9th day from vaccination, and on the 10th, about twenty-four distinct eruptions appeared, none of which maturated.

### Remark on the above.

William had been exposed to variolous contagion, of the most active nature, fifteen days before the second inoculation, and Eliza seven days; being in the same room, the greater part of their time with their brother, and constantly communicating by the attendants on him.

Can this be called, with any regard to truth, a failure of the vaccine inoculation?

Thus do the enemies of the vaccine inoculation, and in these instances, most wickedly, employ every untoward occurrence, whether relative to Cow-pox or not; for, with the help of falsehood, they always make it answer their purpose, to prejudice the minds of the public, particularly the ignorant and weak, against one of the greatest blessings ever bestowed on man; and which it is the blackest ingratitude to the Supreme Being, not to receive with heartfelt and genuine thanks!

Since my last communication, I have vaccinated, (considering the state of vaccination here) a great number; and notwithstanding what Report says to the contrary, I have pleasure in saying, that from the time I commenced vaccination, to the present moment, no failure has occured in my practice. I am, &c.

158, *Queen Street,* SAMUEL HILL, Sen.

*Town of Portsea, Hants.*

---

† William and Eliza,

